# Circ3823 contributes to growth, metastasis and angiogenesis of colorectal cancer: involvement of miR-30c-5p/TCF7 axis

**DOI:** 10.1186/s12943-021-01372-0

**Published:** 2021-06-25

**Authors:** Yaxin Guo, Yuying Guo, Chen Chen, Dandan Fan, Xiaoke Wu, Luyang Zhao, Bo Shao, Zhenqiang Sun, Zhenyu Ji

**Affiliations:** 1 School of Basic Medical Sciences, Academy of Medical Sciences, Henan Institute of Medical and Pharmaceutical Sciences, Zhengzhou University, Zhengzhou, 450001 Henan China; 2 Department of Neurology, The First Affiliated Hospital of Zhengzhou University, Zhengzhou, 450052 Henan China; 3 Department of Colorectal Surgery, The First Affiliated Hospital of Zhengzhou University, Zhengzhou, 450052 Henan China; 4 School of Life Sciences, Zhengzhou University, Zhengzhou, 450001 Henan China

**Keywords:** Colorectal cancer (CRC), Tumour progression, Angiogenesis, circ3823, N6-methyladenosine (m6A)

## Abstract

**Background:**

Colorectal cancer (CRC) is one of the most common malignant tumours. The recurrence and metastasis of CRC seriously affect the survival rate of patients. Angiogenesis is an extremely important cause of tumour growth and metastasis. Circular RNAs (circRNAs) have been emerged as vital regulators for tumour progression. However, the regulatory role, clinical significance and underlying mechanisms still remain largely unknown.

**Methods:**

High-throughput sequencing was used to analyse differential circRNAs expression in tumour and non-tumour tissues of CRC. In situ hybridization (ISH) and qRT-PCR were used to determine the level of circ3823 in CRC tissues and serum samples. Then, functional experiments in vitro and in vivo were performed to investigate the effects of circ3823 on tumour growth, metastasis and angiogenesis in CRC. Sanger sequencing, RNase R and Actinomycin D assay were used to verify the ring structure of circ3823. Mechanistically, dual luciferase reporter assay, fluorescent in situ hybridization (FISH), RNA immunoprecipitation (RIP) and RNA pull-down experiments were performed to confirm the underlying mechanisms of circ3823.

**Results:**

Circ3823 was evidently highly expressed in CRC and high circ3823 expression predicted a worse prognosis of CRC patients. Receiver operating characteristic curves (ROCs) indicated that the expression of circ3823 in serum showed high sensitivity and specificity for detecting CRC which means circ3823 have the potential to be used as diagnostic biomarkers. Functional experiments in vitro *and* in vivo indicated that circ3823 promote CRC cell proliferation, metastasis and angiogenesis. Mechanism analysis showed that circ3823 act as a competing endogenous RNA of miR-30c-5p to relieve the repressive effect of miR-30c-5p on its target TCF7 which upregulates MYC and CCND1, and finally facilitates CRC progression. In addition, we found that N6-methyladenosine (m6A) modification exists on circ3823. And the m6A modification is involved in regulating the degradation of circ3823.

**Conclusions:**

Our findings suggest that circ3823 promotes CRC growth, metastasis and angiogenesis through circ3823/miR-30c-5p/TCF7 axis and it may serve as a new diagnostic marker or target for treatment of CRC patients. In addition, m6A modification is involved in regulating the degradation of circ3823.

**Supplementary Information:**

The online version contains supplementary material available at 10.1186/s12943-021-01372-0.

## Background

Colorectal cancer (CRC) is a common malignant tumour in digestive system, which remains a substantial public health challenge across the globe in the last 30 years [1]. The incidence and mortality rate of CRC are the third and second in all malignant tumours, respectively [2]. A large number of epidemiological data showed that the number of CRC patients were increasing by years in developing and developed countries, and the progress of CRC seriously threatens the survival of patients [3–5]. However, due to the lack of diagnostic biomarkers and therapeutic targets, the treatment of CRC is not ideal and further improvement is needed. Therefore, it is critical to further explore the molecular mechanisms underlying the progression of CRC.

Tumour cells are highly metabolized, and their continuous growth depends on adequate nutrient supply. In the early stage of tumorigenesis, nutrients can penetrate through the tissue to maintain its growth, but when the diameter exceeds 2 mm, the tumour must form new blood vessels to provide nutrition [6–10]. In addition, blood metastasis is one of the main ways of tumour metastasis to distant organs [11, 12]. Therefore, angiogenesis is essential for tumour growth and metastasis. Recently, circRNA has attracted great research interest due to its regulatory role in diseases especially for oncology. Many explorations confirmed that circRNA is closely related to the angiogenesis of CRC [13–15]. The covalently closed loop structure makes circRNA conserved and stable. CircRNA were widely and diversely present in eukaryotic cells, with certain tissue specificity, timing and disease specificity which means it has the potential to be biomarker for tumour diagnosis. The competing endogenous RNA (ceRNA) is the most classic mechanism and have been widely reported in various types of cancer [16–24]. Besides the ceRNA mechanism, RNA-binding proteins and functional proteins coding are main way to function [25–28].

In this study, we initially investigated the function and molecular mechanism of hsa_circ_0001821 (designated as circ3823 through RNA-seq) in CRC, and explored its potential as a diagnostic biomarker. This research revealed that circ3823 promoted the expression of TCF7 and TCF7 downstream MYC and CCND1 via inhibition of miR-30c-5p, resulting in proliferation, metastasis and angiogenesis of CRC. In addition, we found that N6-methyladenosine (m6A) modification exists on circ3823. And the degradation rate of circ3823 was regulated by m6A recognition protein YTHDF3 and demethylase ALKBH5. Our results indicate that circ3823 exerts oncogenic potential and it may be a candidate in diagnosis marker and therapeutic target of CRC.

## Materials and methods

### Tissue, serum and paraffin section sources

Tissue and serum samples which were collected within the last 4 years were obtained from the First Affiliated Hospital of Zhengzhou University, Henan, China. After the tissues and serums were separated from the human body, they were quickly transferred to liquid nitrogen for storage. Since the tissue and serum samples were not repeatedly frozen and thawed, the experimental data truly reflected the RNA level in the body.

Paraffin-embedded tissue sections were also obtained from the First Affiliated Hospital of Zhengzhou University. Samples from patients with CRC treated between 2012 and 2016 were selected. This study was approved by the Ethics Committee of the First Affiliated Hospital of Zhengzhou University, and all patients signed informed consent forms.

### RNA isolation, library synthesis and RNA sequencing

Total RNA was isolated and purified using Trizol reagent (Invitrogen, Carlsbad, USA) following the manufacturer’s procedure. The RNA amount and purity of each sample was quantified using NanoDrop ND-1000 (NanoDrop, Wilmington, USA). The RNA integrity was assessed by Agilent 2100 with RIN number > 7.0. Approximately 5 μg of total RNA was used to deplete ribosomal RNA according to the manuscript of the Ribo-Zero rRNA Removal Kit (Illumina, San Diego, USA). After removing ribosomal RNAs, the left RNAs were fragmented into small pieces using divalent cations under high temperature. Then the cleaved RNA fragments were reverse-transcribed to create the cDNA, which were next used to synthesise U-labeled second-stranded DNAs with *E. coli* DNA polymerase I, RNase H and dUTP. An A-base is then added to the blunt ends of each strand, preparing them for ligation to the indexed adapters. Each adapter contains a T-base overhang for ligating the adapter to the A-tailed fragmented DNA. Single-or dual-index adapters are ligated to the fragments, and size selection was performed with AMPureXP beads. After the heat-labile UDG enzyme treatment of the U-labeled second-stranded DNAs, the ligated products are amplified with PCR by the following conditions: initial denaturation at 95 °C for 3 min; 8 cycles of denaturation at 98 °C for 15 s, annealing at 60 °C for 15 s, and extension at 72 °C for 30 s; and then final extension at 72 °C for 5 min. The average insert size for the final cDNA library was 300 bp (±50 bp). At last, we performed the paired-end sequencing on an Illumina Hiseq 4000 (LC Bio, China) following the vendor’s recommended protocol.

### Cell culture

HCT116 cells were obtained from iCell Bioscience Inc. (Shanghai, China). SW480, DLD-1 and HT29 cells were obtained from the Biotherapy Center of The First Affiliated Hospital of Zhengzhou University. All cells were cultured in DMEM/high-glucose (HyClone, Logan, Australia) with 10% fetal bovine serum (Biological Industries, Cromwell, USA) at 37 °C and 5% CO2 under saturated humidity.

### Total RNA isolation and real-time quantitative PCR (qRT-PCR) assay

Total RNA was isolated from serum and tissues using RNAiso Plus (Takara, Dalian, China) according to the manufacturer’s instructions. The integrity and purity of the extracted total RNA were measured using NanoDrop One (Thermo Fisher Scientific, Waltham, USA) ultra-micro UV spectrophotometer. Reverse transcription was performed using the PrimeScript RT reagent Kit (Takara, Dalian, China) with gDNA Eraser. After removing the genomic DNA at 42 °C for 2 min, the tissue RNA was reverse transcribed into cDNA under the following conditions: 37 °C for 15 min and 85 °C for 5 s. Serum RNA was reverse transcribed into cDNA using a RevertAid H Minus First Strand cDNA Synthesis Kit (Thermo Fisher Scientific, Waltham, USA) under the following conditions: 25 °C for 5 min, 42 °C for 60 min, and 70 °C for 5 min. The product was immediately stored at − 80 °C until use.

The qRT-PCR was performed on a QuantStudio 5 Real-Time PCR System (Applied Biosystems, Foster City, USA) using a Hieff qPCR SYBR Green Master Mix kit (Yeasen, Shanghai, China). The qRT-PCR reaction was performed 95 °C for 5 min, followed by 40 cycles of 95 °C for 10 s and a primer-specific annealing temperature of 60 °C for 30 s. The qRT-PCR primer sequences were provided in Table S2. The relative quantification values for RNA were calculated by the 2^−ΔΔCt^ method using GAPDH as an internal reference.

### Vector construction and cell transfection

The full-length of circ3823 was cloned into over expression vector pcDNA3.1 (Hanbio Biotechnology, Wuhan, China), while the mock vector with no circ3823 sequence served as a control. SiRNAs targeting the back-splice junction site of circ3823 and siRNA-NC were synthesized by RiboBio (RiboBio, Guangzhou, China), efficiency detected by qRT-PCR. The mimics and inhibitors of miR-30c-5p were purchased from RiboBio (RiboBio, Guangzhou, China). Lipofectamine 3000 (Invitrogen, Carlsbad, USA) were used to cell transfections. The sequences of siRNAs were listed in Table S5.

### Western blot analysis

Total protein of CRC tissues or cell lines were extracted by RIPA with PMSF and determined via BCA Protein Assay Kit (Solarbio, Beijing, China). Equal amounts of protein were separated on SDS-PAGE gels and then transferred to PVDF membrane (Millipore, Massachusetts, USA). After blocked with 5% BSA in TBST, PVDF membrane were incubated with primary antibodies against CCND1 (1:1000), MYC (1:1000) (Abways, Shanghai, China), TCF7 (1:500) (Santa Cruz, CA, USA) and GAPDH (1:5000) (Proteintech, Wuhan, China) at 4 °C overnight and then hybridized with a secondary antibody at 37 °C for 1 h. The intensity of the bands was analysed with a chemiluminescence kit (Millipore, Massachusetts, USA).

### In situ hybridization

The in-situ hybridization probes and kits were designed and synthesized by Wuhan Servicebio company. The probe sequences were listed in Table S4. After prehybridization at 37 °C for 2 h, tissue sections were hybridized with specific DIG-labelled circ3823 probes at 37 °C overnight, and stained by DAB and hematoxylin (Solarbio, Beijing, China). Slides were photographed with a fluorescence microscope (Olympus, Tokyo, Japan). ISH was independently evaluated at 200× magnification using light microscopy by two pathologists who were blinded to the clinicopathological data. A semiquantitative evaluation of circ3823 was performed using a method described in the previous works [23, 29]. The evaluation procedure was based on staining intensity and extent of staining as follows: staining intensity for circ3823 was scored as 0 (negative), 1 (weak), 2 (moderate) and 3 (strong). Staining extent was scored as 0 (0), 1 (1–25%), 2 (26–50%), 3 (51–75%) and 4 (76–100%), depending on the percentage of positively stained cells. The product of the staining intensity and the staining extent scores were regarded as ISH scores. The agreement between the two evaluators was 90%, and all scoring discrepancies were resolved by discussion between the two evaluators.

### RNase R treatment

Total RNA (2 μg/group) of SW480 cells were incubated for 0 min, 10 min, 20 min, 30 min at 37 °C with 5 U/μg RNase R (Epicentre Technologies, Madison, USA), and subsequently the abundance of linear RNA and circular RNA were analysed by qRT-PCR.

### Actinomycin D assay

SW480 cells were exposed to 100 ng/ml actinomycin D (Merck, Darmstadt, Germany) at 0 h, 4 h, 8 h, 12 h, 24 h. Then the cells were harvested, and total RNA was extracted. The stability of circ3823 and PVT1 mRNA were analysed using qRT-PCR.

### Nuclear and cytoplasmic extraction and fluorescence in situ hybridization (FISH)

The nuclear and cytoplasmic fractions of RNA were extracted with a PARIS™ kit (Invitrogen, Thermo Fisher Scientific, Waltham, USA). Place the cell slide at the bottom of the 24-well plate and cultivate an appropriate number of cells (6 × 10^4^/well). Before the experiment, make the cell confluence reach 60–70%. FISH assay was executed to observe the location of circ3823 and miR-30c-5p in CRC cells. Briefly, after prehybridization at 37 °C for 30 min, cell climbing piece were hybridized with 2.5 μL 20 μM specific Cy3-labelled circ3823 probes and FAM-labelled miR-30c-5p probes (Servicebio, Wuhan, China) at 37 °C overnight, and dyed with DAPI. The probe sequences were shown in Table S4. Slides were photographed with confocal laser scanning microscopy (Zeiss, Jena, Germany).

### CCK-8, EDU, Colony formation, apoptosis and tube junction forming of HUVEC assays

Cell Counting Kit-8 (Dojindo Laboratories, Kumamoto, Japan), Cell-Light EdU Apollo567 In Vitro Kit (RiboBio, Guangzhou, China) and Colony formation assays were used to detect proliferation of CRC cells according to the manufacturer’s instructions. HCT116 cell apoptosis was detected by Annexin V-FITC/propidium iodide (PI) apoptosis detection kit (Beyotime, Shanghai, China). The cells were harvested and double stained with FITC and PI after transfection, followed analysed on a flow cytometer (Becon Dickinson FACSCalibur, NY, USA). As for tube junction forming assay of HUVEC, the culture supernatants of HCT116 and SW480 cells transfected with plasmid or siRNA were collected, followed incubate with 3 × 10^4^ HUVEC cells per well in a 96-well culture plate precoated with matrix (Corning, NY, USA) for 6 h. Finally, the tube junction formation was observed under an optical microscope (Olympus, Tokyo, Japan).

### Migration and invasion assays

After transfection, 2 × 10^4^ CRC cells were seeded into the upper chambers without Matrigel (Corning, NY, USA), and 500 μL complete medium was added into the bottom chambers (Corning, NY, USA) for migration assays. For invasion assays, 1 × 10^5^ CRC modified cells were seeded into the upper chambers with Matrigel (Corning, NY, USA). After 36 h, the cells on the compartment were fixed in 4% paraformaldehyde (Beyotime, Shanghai, China) and stained by crystal violet (Solarbio, Beijing, China), then photographed and counted with an optical microscope (Olympus, Tokyo, Japan).

### Animal experiments

Female BALB/c nude mice (4-week-old) were housed under standard conditions. In the tumour growth xenograft model, 5 × 10^6^ HCT116 cells with LV-circ3823 or LV-NC were suspended in 100 μL serum-free DMEM and subcutaneously injected into the right flank of each mouse. The volumes of tumours were measured every 3 days and calculated as 0.5 × length×width^2^. After 30 days the mice were sacrificed and the tumours were removed for further analysis. In the “tail vein–lung metastasis” nude mouse models, 2.5 × 10^6^ HCT116 cells with LV-circ3823 or LV-NC were injected into the tail vein of mice. For imaging tumours in live animals, D-Luciferin, Potassium Salt D (Yeasen, Shanghai, China) was dissolved in sterile distilled water (final concentration: 15 mg/ml). Mice were anaesthetized with isoflurane and injected intraperitoneally with 100 μl of the luciferin solution. After 10 mins, images were acquired with the IVIS Lumina series III (PerkinElmer, Waltham, Massachusetts, USA). The mice were killed after 49 days, and all the lungs were surgically removed.

### Bioinformatics prediction of the circRNA-miRNA-mRNA network

The competing endogenous RNA (ceRNA) hypothesis reveals an interaction mechanism between RNAs. We predicted the target microRNAs of circ3823 with TargetScan and regRNA. More reliable microRNAs were screened based on the intersection of TargetScan (http://www.targetscan.org/vert_72/) and RegRNA 2.0 (http://regrna2.mbc.nctu.edu.tw/). The target mRNA of each microRNA was predicted by TargetScan.

### Immunohistochemistry (IHC) and immunofluorescence (IF)

For IHC assay, paraffin sections were incubated with primary antibodies against TCF7 (1:50) (Santa Cruz, CA, USA), MYC (1:100), CCND1 (1:100) (Abways, Shanghai, China), CD34 (1:800), CD31(1:1500), α-SMA (1:2000), collagen IV (1:1000) and ki67 (1:10000) (Proteintech, Wuhan, China) at 37 °C for 60 mins, secondary antibodies at 37 °C for 15 mins and horseradish enzyme labelled streptavidin solution for 10 min, then stained by DAB and hematoxylin. For IF analysis, cell climbing pieces were incubated with TCF7 antibodies overnight at 4 °C and AF488-conjugated secondary antibodies (Biolegend, San Diego, USA), then dyed by DAPI and observed by a fluorescence microscope (Olympus, Tokyo, Japan).

### Luciferase activity assays

The sequences of circ3823, TCF7–3’UTR and their miR-30c-5p binding sites mutant versions were synthesized and add to luciferase reporter vector psiCHECK2 (Hanbio Biotechnology, Wuhan, China), named circ3823-WT, circ3823-Mut, TCF7-WT and TCF7-Mut, respectively. The experimental steps follow the manufacturer’s protocols of Dual Luciferase Assay Kit (Hanbio Biotechnology, Wuhan, China). The relative luciferase activity was examined by Full-wavelength multifunctional enzyme label tester SpectraMax M5e (Molecular Devices, Shanghai, China).

### RNA immunoprecipitation (RIP)

According to the manufacturer’s instructions of Magna RIP RNA-Binding Protein Immunoprecipitation Kit (Millipore), RIP experiments were conducted with AGO2 antibody (Abcam, Burlingame, USA) and anti-m6A antibody (Synaptic Systems, Goettingen, Goettingen). Co-precipitated circ3823 was applied to qRT-PCR.

### Biotin-coupled miRNA capture

The 5′ biotinylated miR-30c-5p pulldown probe or NC pulldown probe were designed and synthesized by Genepharma (Shanghai, China). HCT116 cells were lysed with RIPA and PMSF (Solarbio, Beijing, China) and incubated with miR-30c-5p probes or NC probe. Then cell lysates were incubated with streptavidin-coated magnetic beads (Invitrogen, Thermo Fisher Scientific, Waltham, USA) to pull down the biotin-labelled RNA complex. The RNA was purified with TRIzol (Takara, Dalian, China). Then the abundance of circ3823 and TCF7 was analysed by qRT-PCR.

### Statistical analysis

All findings were shown as mean ± standard deviation (SD) and analysed with IBM SPSS Statistics 21 (Chicago, IL, United States), and graphs were generated by GraphPad Prism 7.0. Continuous data analysis was performed with Student’s t-test to analyse differential expression between two groups. ROC curves were used to assess the sensitivity and specificity of circRNA as a diagnostic biomarker. Survival curves were compared with the log-rank test. All statistical tests were 2 sided, and *P* <  0.05 was considered statistically significant.

## Results

### Circ3823 was highly expressed in colorectal cancer (CRC)

**Fig. 1:**
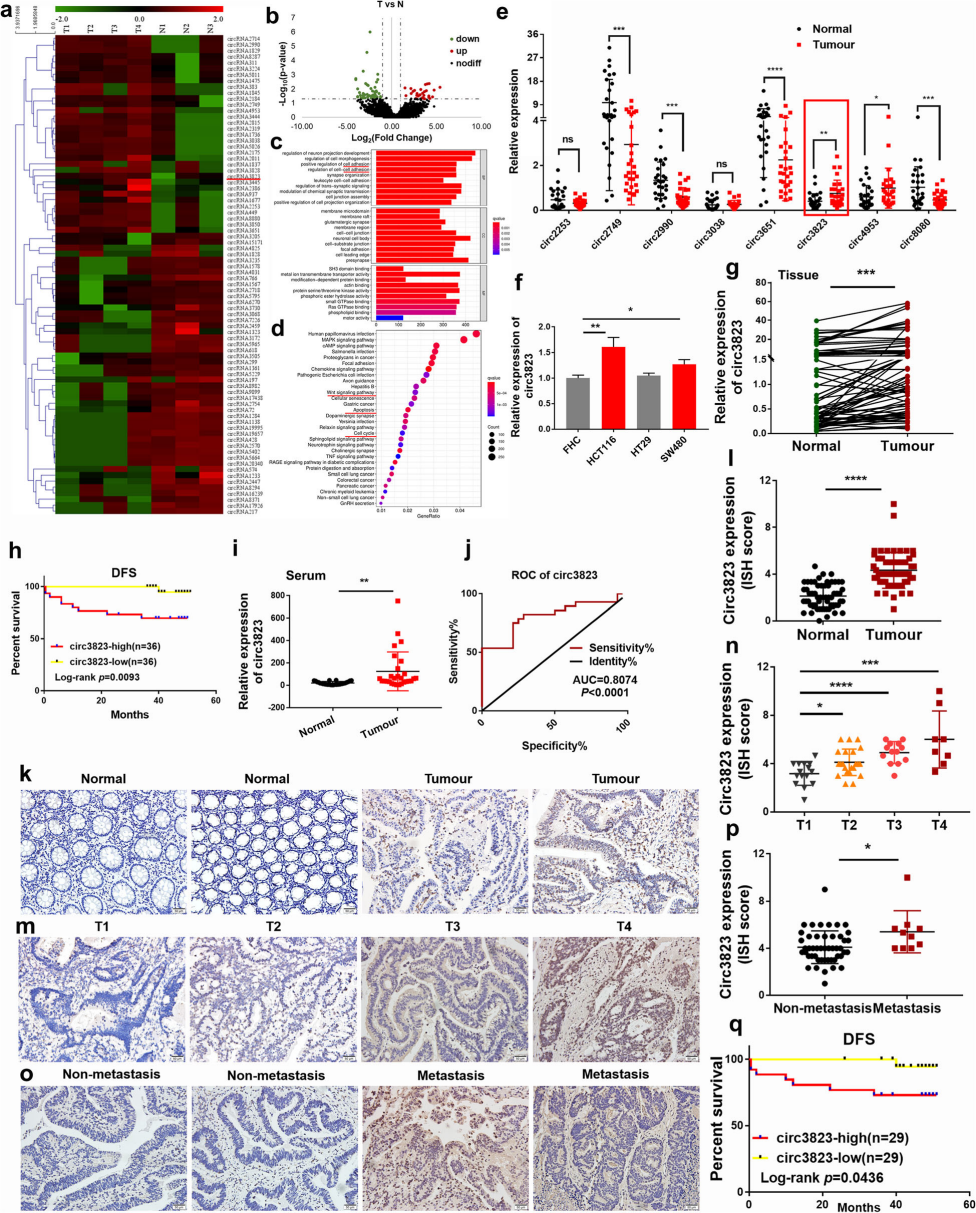
CircRNA expression profiling in CRC tissues and normal tissues and validation of the circRNAs expression in serums and tissues (T: tumour, N: normal). **a** Hierarchical clustering of differentially expressed circRNA in two groups. **b** Volcano plot showed the expression profile between tumour tissue and normal tissue. Conditions for screening differences:│Fold Change│ > 2; *p* < 0.05. The red points in the plot indicated significantly up-regulated circRNAs, and the green points indicated significantly down-regulated circRNAs. **c** GO function analysis of differentially expressed circRNAs. **d** KEGG pathway analysis of differentially expressed circRNAs. **e** The expression of circ2253, circ2749, circ2990, circ3038, circ3651, circ3823, circ4953 and circ8080 was detected by qRT-PCR in 28 CRC tissues and matched normal tissues. **f** The expression of circ3823 was measured by qRT-PCR in CRC cells and FHC. **g** The level of circ3823 in 72 CRC tissues and matched normal tissues were analyzed by qRT-PCR. **h** The disease-free survival analysis of 72 CRC patients was performed based on qRT-PCR data. Log-rank test was used to estimate the significance. **i** The expression of circ3823 was measured by qRT-PCR in 28 serum samples from CRC patients and 28 serum samples from healthy people. GAPDH was used as an internal reference. **j** ROC curves based on the expression of circ3823 in serum. **k, l** ISH images and ISH scores statistical analysis of circ3823 in CRC tissues and normal tissues. **m, n** ISH images and ISH scores statistical analysis of circ3823 in T1, T2, T3, T4 CRC tissues. **o, p** ISH images and ISH scores statistical analysis of circ3823 in lymph node metastasis tissues and non-lymph node metastasis tissues (magnification, × 200, scale bar, 50 μm). **q** The disease-free survival analysis of 58 CRC patients based on circ3823 ISH scores. Log-rank test was used to estimate the significance. Data were indicated as mean ± SD, ns *P* ≥ 0.05, *P < 0.05, **P < 0.01, ***P < 0.001, ****P < 0.0001

In order to understand the expression profiles of circRNA in CRC, CRC tissues and para-cancerous tissues were constructed by RNA-seq. Hierarchical clustering indicated differences in circRNA expression profiling between the two groups (Fig. 1a). There were 80 differentially expressed circRNAs with a cut-off criteria of fold change > 2.0 and *P* <  0.05, including 33 up-regulated circRNAs and 47 down-regulated circRNAs (Fig. 1b). Moreover, 5332 differentially expressed mRNAs were obtained, including 3525 up-regulated and 1807 down-regulated genes. GO functional annotation and KEGG enrichment showed differentially expressed genes were mainly involved in phenotype of CRC (Fig. 1c, d). Then eight upregulated RNAs (circ2253, circ2749, circ2990, circ3038, circ3651, circ3823, circ4953 and circ8080) were selected for further analysis due to their host gene function. Preliminarily, the expression of 8 selected circRNAs were verified in 28 tissue pairs by qRT-PCR (Fig. 1e). Notably, circ3823 (hsa_circ_0001821) was the most significant circRNA, which was spliced from PVT1 located at chr8: 127890588–127,890,998 and finally formed a circular transcript of 410 nt according to the annotation of circBase (http://www.circbase.org/). Interestingly, circ3823 was also markedly upregulated in CRC cell lines (HCT116, HT29, SW480) compared with normal cell line (FHC) (Fig. 1f). In addition, the expression of circ3823 from 72 CRC tissues was detected by qRT-PCR (Fig. 1g). Then the 72 CRC patients were divided into high circ3823 expression group (*n* = 36) and low circ3823 expression group (*n* = 36) based on the median. The results showed that the group of high circ3823 expression predicted a worse prognosis of CRC patients (Fig. 1h).

Interestingly, there was also a sharp increase of circ3823 expression in the serum of CRC patients (Fig. 1i). ROC curve analysis was performed based on the expression of circ3823 in serum. The AUC was 0.8074 (95% CI: 0.6900–0.9248, *p* < 0.0001) (Fig. 1j). These results suggest that circ3823 in serum has good sensitivity and specificity as diagnostic marker for CRC. To further verify the expression of circ3823 in CRC tissue, we performed ISH on paraffin-embedded tissue sections which containing 58 CRC tissues and 58 normal tissues. ISH images and scores showed that the expression of circ3823 in CRC tissues was higher than that in normal tissues (Fig. 1k, l). Moreover, high expression of circ3823 in CRC tissues was significantly correlated with tumour differentiation status, depth of bowel wall invasion and lymph node metastasis (Fig. 1m, n, o, p) (Table 1). In addition, the disease-free survival analysis based on ISH scores indicated that the group of high circ3823 expression predicted a worse prognosis of CRC patients (Fig. 1q).

**Table 1:** Correlation analysis for clinicopathologic parameters about circ3823 expression in colorectal cancer patients (*χ*^2^ test)

Parameters	Number of cases	Circ3823 expression		***P*** value
		Low	High	
All cases	58	29	29	
**Age**				0.599
< 61	28	13	15	
≥ 61	30	16	14	
**Gender**				0.426
Male	33	18	15	
Female	25	11	14	
**Histological differentiation**				0.015
Moderately differentiated	48	28	20	
Poorly differentiated	10	1	9	
**Tumour depth**				< 0.001
T1-T2	37	27	10	
T3-T4	21	2	19	
**Lymph node metastasis**				0.001
Yes	10	0	10	
No	48	29	19	
**CEA**				0.139
< 2.06	28	17	11	
≥ 2.06	27	11	16	

### Identification of the ring structure of circ3823 and its cellular location

**Fig. 2:**
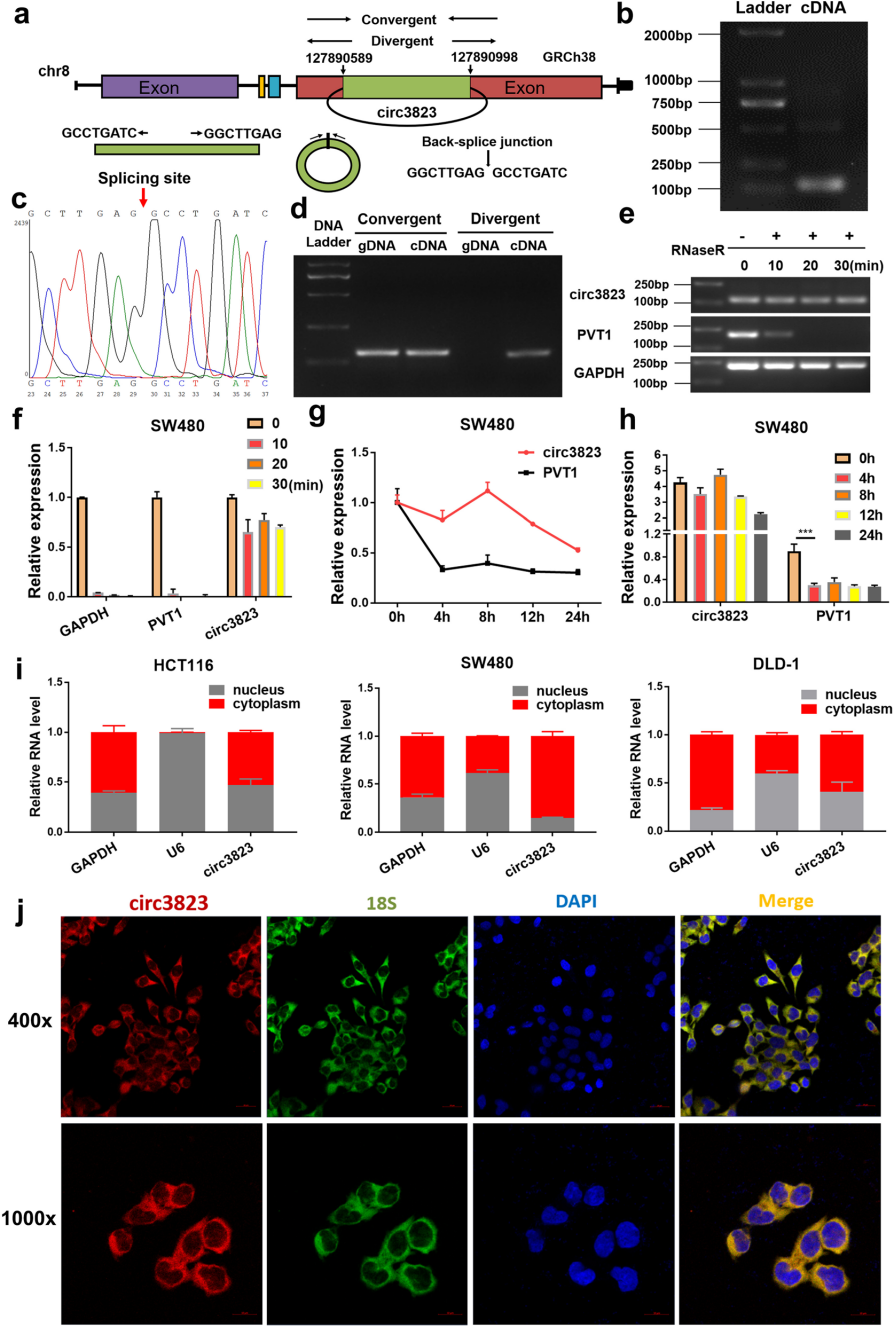
Verification of the closed loop structure of circ3823 and its subcellular location. **a** The formation process of circ3823 which was produced at the PVT1 gene located on chromosome8. **b** PCR product of circ3823 in agarose gel electrophoresis. **c** The back-splice junction of circ3823 was identified by Sanger sequencing. **d** Convergent and divergent primers were used to verification of the closed loop structure. **e, f** Verification of the closed loop structure of circ3823 through RNase R. **g, h** The abundance of circ3823 and PVT1 were detected by qRT-PCR in SW480 cells treated with Actinomycin D at the indicated time point. **i** qRT-PCR was used to measure the level of circ3823 in the nuclear and cytoplasmic of HCT116, SW480, DLD-1 cells. **j** FISH was performed to observe the cellular location of circ3823 (red) and 18S (green) in cells (magnification, × 400, scale bar, 20 μm and magnification, × 1000, scale bar, 10 μm). Data were indicated as mean ± SD (*n* = 3), ns *P* ≥ 0.05, *P < 0.05, **P < 0.01, ***P < 0.001, ****P < 0.0001

The splicing process model diagram of circ3823 was shown in Fig. 2a. To verify the closed loop structure of circ3823, convergent and divergent primers were designed for circ3823 and corresponding PVT1 linear transcript (Table S3). The back-spliced junction of circ3823 was amplified using divergent primers and confirmed by Sanger sequencing (Fig. 2b, c). The sequence is consistent with RNAseq and circBase database annotation. In SW480 cells, circ3823 could be amplified from only cDNA but not gDNA (Fig. 2d). Furthermore, circ3823 was more stable than PVT1 in SW480 cells treated with RNase R and Actinomycin D (an inhibitor of transcription) (Fig. 2e, f, g, h). To observe cellular localization of circ3823, qRT-PCR analysis was conducted for nuclear and cytoplasmic circ3823. Results showed that circ3823 preferentially located in the cytoplasm (Fig. 2i). In addition, FISH assay also showed that most of circ3823 (red) located in cytoplasm in SW480 cells (Fig. 2j).

### Effects of circ3823 on cell proliferation, apoptosis, migration, invasion and angiogenesis in vitro

**Fig. 3:**
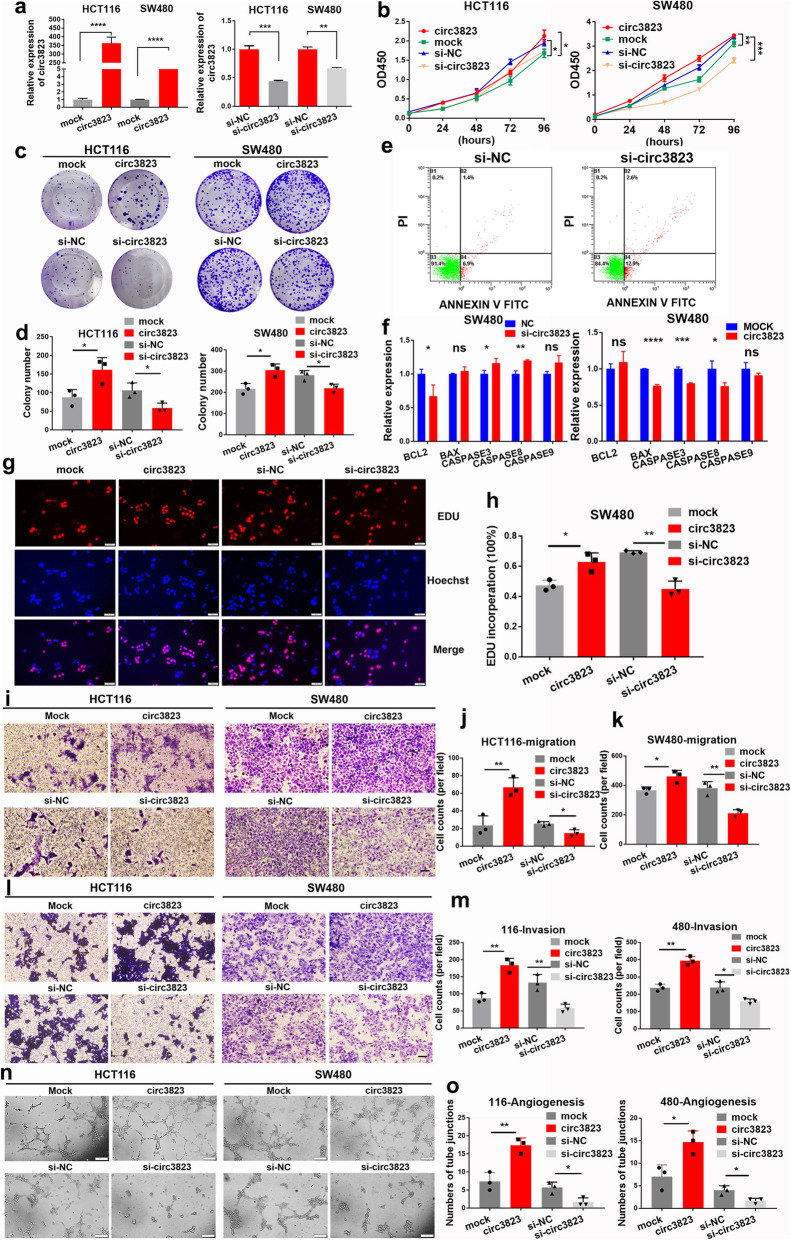
Circ3823 promotes CRC cell proliferation, migration, invasion and increases the tube junction forming ability of HUVEC in vitro. **a** Expression levels of circ3823 in HCT116 and SW480 cells treated with circ3823 plasmid and circ3823 siRNA. **b, c, d, g, h** Cell proliferation detection of CRC cells were measured by CCK-8, clone formation and EDU assay (magnification, × 200, scale bar, 50 μm). **e** 1 × 10^4^ SW480 cells were counted per experiment to determine apoptosis rate by flow cytometry via Annexin V-FITC/PI. **f** Apoptosis-related molecules were detected using qRT-PCR in SW480 cells. **i, j, k, l, m** Cell migration and invasion abilities were determined by transwell assays (magnification, × 100, scale bar, 100 μm). **n, o** Tube junction forming ability of HUVEC were determined by incubate with HCT116, SW480 cell culture supernatant after transfection (magnification, × 100, scale bar, 200 μm). Data were indicated as mean ± SD, ns *P* ≥ 0.05, *P < 0.05, **P < 0.01, ***P < 0.001, ****P < 0.0001

To explore the biological function of circ3823 in CRC cells, the overexpression vector and the siRNA against circ3823 were constructed. As shown in Fig. 3a, the overexpression vector and the siRNA of circ3823 were efficiently increased and reduced the expression of circ3823 in HCT116 and SW480 cells, respectively. CCK8 assays was performed to draw growth curves which demonstrated that upregulation of circ3823 significantly enhanced the proliferation viability of HCT116 and SW480 cells, whereas downregulation of circ3823 inhibited cell growth (Fig. 3b). Colony formation assays further demonstrated that the cell colony number of HCT116 and SW480 were significantly increased by upregulation of circ3823 and markedly impaired by downregulation of circ3823 (Fig. 3c, d). Similarly, EdU assays revealed that overexpression of circ3823 markedly increased the percentages of EdU-positive cells, while knockdown of circ3823 decreased the ratio of positive cells (Fig. 3g, h). These results suggested that circ3823 enhances proliferation of CRC cells.

Then, the effects of circ3823 on migration and invasion of CRC cells was further evaluated using trans well assays. As shown in Fig. 3i, j, k, l, m, overexpression and knockdown of circ3823 markedly increased or reduced migration and invasion abilities in HCT116 and SW480 cells, respectively. In addition, compared to NC group, apoptosis rate was clearly increased in SW480 cells transfected with si-circ3823 (Fig. 3e). Similarly, knockdown of circ3823 was clearly promoted expression of apoptosis-related factor (*Bax, caspase3, caspase8, caspase9*) and decreased expression of anti-apoptotic factor *Bcl-2* by qRT-PCR in SW480 cells (Fig. 3f). Simultaneously, higher expression of *Bcl2* and lower expression of *Bax, caspase3, caspase8, caspase9* were found in overexpression circ3823 group when compared to mock group (Fig. 3f). Furthermore, overexpression and knockdown of circ3823 markedly increased or reduced the ability of tube junction formation of HUVEC (Fig. 3n, o).

### Circ3823 promotes proliferation, metastasis and angiogenesis in vivo

**Fig. 4:**
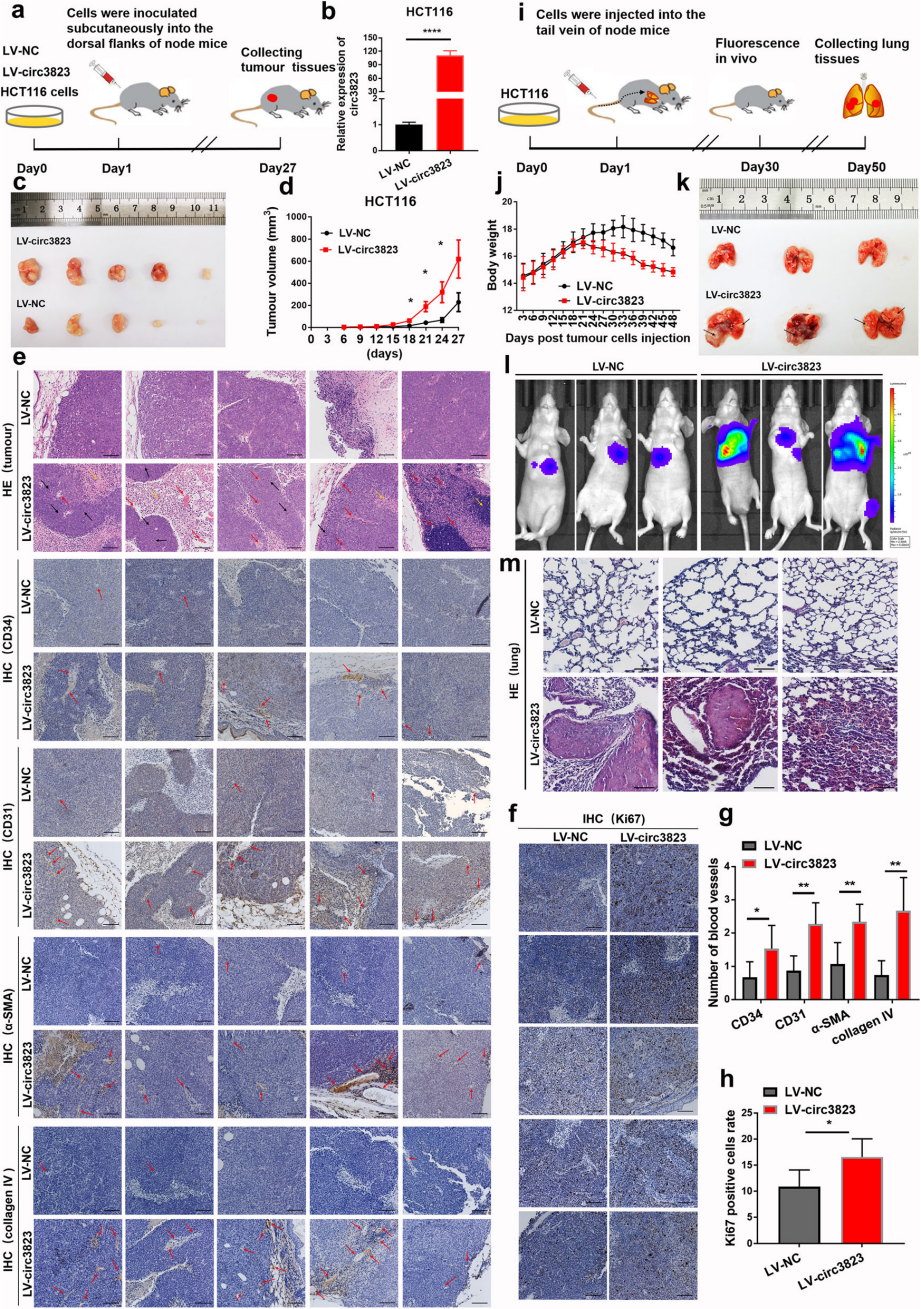
Circ3823 facilitates tumourigenesis, metastasis and angiogenesis in vivo. **a, b** The process of constructing subcutaneous xenograft tumour in nude mice. CRC cell line stably overexpressing circ3823 was constructed by lentivirus and the expression of circ3823 was detected by qRT-PCR. **c** Images of xenograft tumours of each group (*n* = 5). **d** Growth curves of xenograft tumours measured every 3 days. **e, g** Pathology of the tumour tissue was detected by HE staining. IHC staining of CD34/31, α-SMA and collagen IV to investigate angiogenesis (mitotic phase: black arrow, angiogenesis: red arrow, inflammatory cell infiltration: yellow arrow) (magnification, × 100, scale bar, 100 μm). **f, h** IHC staining of ki-67 in each slide to evaluate cell cycle (magnification, × 100, scale bar, 100 μm). **i** The process of constructing metastasis xenograft tumour in nude mice. **j** The body weight of the two groups of nude mice. **k** After injection of stably overexpressing circ3823 HCT116 cells into the tail vein of nude mice at 50 days, the mice were euthanized, and metastatic lung nodules were marked with black arrow. **l** Bioluminescent images of lungs for each experimental group at 7 weeks. **m** HE staining of lung sections displayed metastatic nodules of the lungs (magnification, × 100, scale bar, 100 μm). Data were indicated as mean ± SD, ns *P* ≥ 0.05, *P < 0.05, **P < 0.01, ***P < 0.001, ****P < 0.0001

To determine the effects of circ3823 on tumour growth, metastasis and angiogenesis in vivo, stably transfected HCT116 cells infected with LV-NC or LV-circ3823 were constructed and subcutaneously injected into 4 weeks old female nude mice (Fig. 4a, b). Importantly, the tumour volumes in the LV-circ3823 group were significantly larger compared to the LV-NC group (Fig. 4c, d). Moreover, hematoxylin eosin (HE) staining and IHC of CD34/31, α-SMA, collagen IV and ki67 showed that upregulation of circ3823 significantly increased the mitotic phase (black arrow), angiogenesis (red arrow) and inflammatory cell infiltration (yellow arrow) (Fig. 4e, f, g, h).

In addition, stably transfected cells were injected into the blood circulation of nude mice through the tail vein (Fig. 4i). As shown in Fig. 4j, the body weight of nude mice in overexpression circ3823 group was significantly decreased after 21 days, compared with the control group. Interestingly, after exposed to HCT116 cells for 7 weeks, the fluorescence intensity of metastatic lung nodules in mice were stronger in LV-circ3823 group (Fig. 4l). Similarly, more nodules were observed in the lungs of overexpression circ3823 group (Fig. 4k). HE staining results showed that overexpression of circ3823 increased quantity and volume of metastasis nodes in the lungs of nude mice (Fig. 4m).

### Circ3823 functions as a sponge for miR-30c-5p

**Fig. 5:**
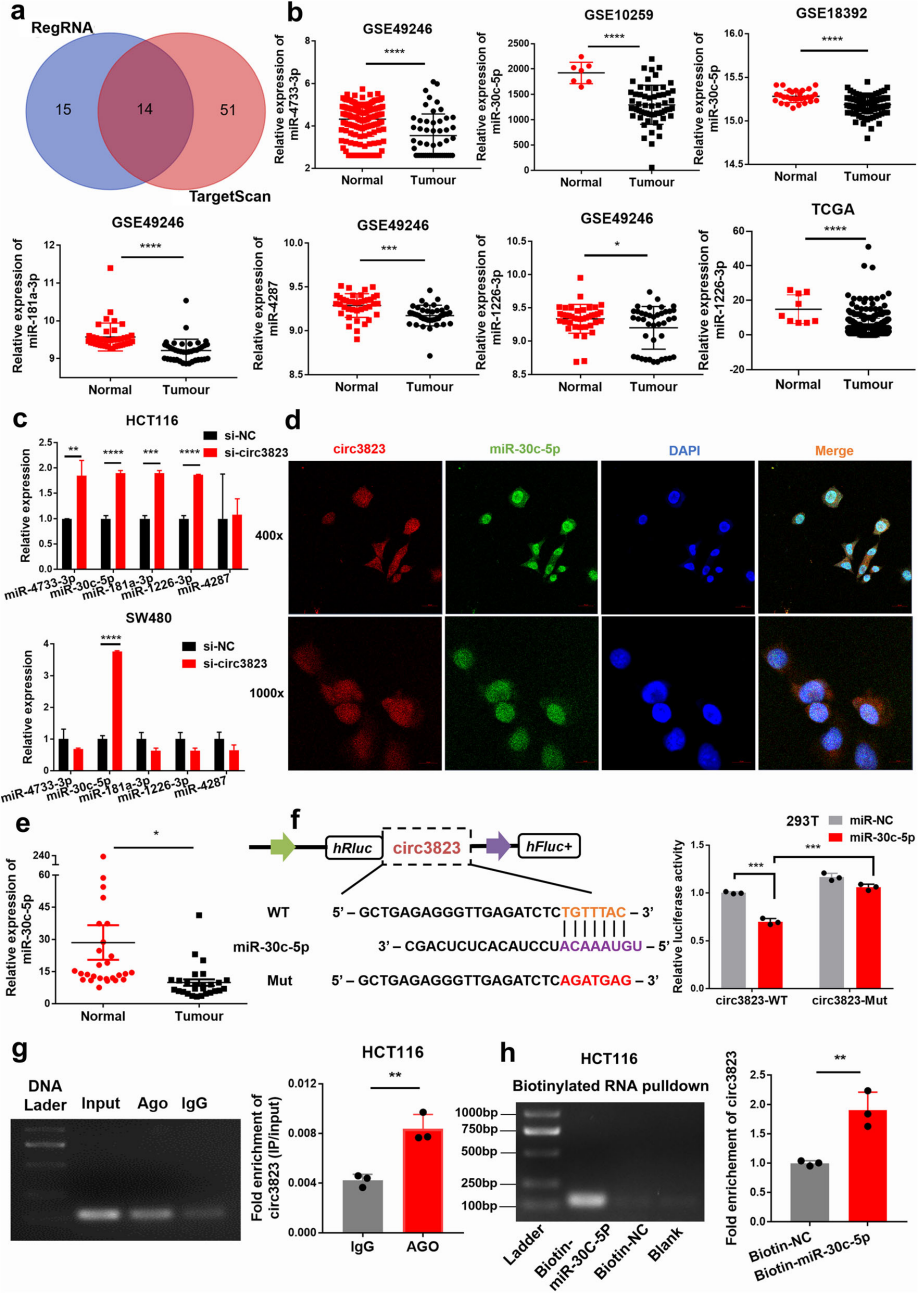
Circ3823 functions as a sponge for miR-30c-5p. **a** The microRNA binding on circ3823 predicted by targetScan and RegRNA. **b** MiR-4733-3p, miR-30c-5p, miR-181a-3p, miR-4287 and miR-1226-3p was significantly down-regulated in CRC according to GEO and TCGA. **c** MiR-30c-5p was detected using qRT-PCR after downregulation of circ3823 in HCT116 and SW480. **d** FISH was performed to observe the cellular location of circ3823 (red) and miR-30c-5p (green) in cells (magnification, × 400, scale bar, 20 μm and magnification, × 1000, scale bar, 10 μm). **e** Relative expression of miR-30c-5p in CRC tissues and adjacent non-tumour tissues was determined by qRT-PCR (*n* = 27). **f** Schematic illustration of circ3823-WT and circ3823-Mut luciferase reporter vectors. The relative luciferase activities were detected in 293 T cells after transfection with circ3823-WT or circ3823-Mut and miR-30c-5p mimics or mimic-NC, respectively. **g** Anti-AGO2 RIP was executed in HCT116 cells, followed by qRT-PCR and nucleic acid electrophoresis to detect the enrichment ability of AGO2 on circ3823 compared with IgG. **h** RNA pull-down was executed in HCT116 cells, followed by qRT-PCR and nucleic acid electrophoresis to detect the enrichment of circ3823. Data were indicated as mean ± SD, ns *P* ≥ 0.05, *P < 0.05, **P < 0.01, ***P < 0.001, ****P < 0.0001

To elucidate the molecular mechanism underlying circ3823, firstly, we predicted the potential targets of circ3823 by miRNA target prediction software TargetScan and regRNA. The intersection of these two databases screened 14 highly reliable microRNAs (Fig. 5a). Then, we use Gene Expression Omnibus (GEO, https://www.ncbi.nlm.nih.gov/geo) and The Cancer Genome Atlas (TCGA, https://cancergenome.nih.gov/) databases to check whether these 14 microRNAs were significantly down-regulated in CRC. After analysis, it was found that miR-4733-3p, miR-30c-5p, miR-181a-3p, miR-4287 and miR-1226-3p was significantly down-regulated in CRC (Fig. 5b). The remaining 9 microRNAs were up-regulated in CRC or had no significant difference. Therefore, these 5 significantly down-regulated microRNAs were regarded as the focus of attention. Next, after interfering with circ3823 in HCT116 and SW480 cells, miR-30c-5p was significantly up-regulated in both cell lines (Fig. 5c). Considering that circ3823 could serve as miRNA sponges in the cytoplasm, we performed FISH assay in SW480 cells to observe the subcellular localization of circ3823 and miR-30c-5p. It was found that circ3823 (red) and miR-30c-5p (green) were co-located in cytoplasm (Fig. 5d). Then, the level of miR-30c-5p was examined in 27 pairs of CRC tissues and adjacent non-cancerous tissues, the results indicated that miR-30c-5p was markedly downregulated in CRC tissues compared with adjacent non-tumour tissues (Fig. 5e). Therefore, we supposed that circ3823 might serve as a ceRNA for miR-30c-5p.

In order to confirm the above prediction analysis, dual-luciferase reporter assay was applied in 293 T cells. The full-length of circ3823-WT and mutant version without miR-30c-5p binding sites were subcloned into luciferase reporter vector psiCHECK2 (Fig. 5f). The results indicated that miR-30c-5p mimics significantly decrease the luciferase activity of WT group but not mutant group (Fig. 5f), suggesting that there might be a direct interaction between circ3823 and miR-30c-5p.

It has been widely known that the key component of RNA-induced silencing complex (RISC) is Argonaute2 (AGO2). Which is necessary for miRNAs regulate target gene expression. Thus, an anti-AGO2 RNA immunoprecipitation (RIP) assay was conducted in HCT116 cell. AGO2 antibody as training group to pull down the RNA transcripts which bind to AGO2, and IgG as negative control. Indeed, circ3823 were efficiently pulled down by anti-AGO2 antibodies compared with IgG (Fig. 5g). To further verify the binding of circ3823 and miR-30c-5p, we performed a microRNA pull-down assay with specific biotin-labelled miR-30c-5p probe. Interestingly, a specific enrichment of circ3823 was detected by qRT-PCR in the miR-30c-5p probe group compared with control probe (Fig. 5h).

### Circ3823 targets miR-30c-5p to promote TCF7 expression

**Fig. 6:**
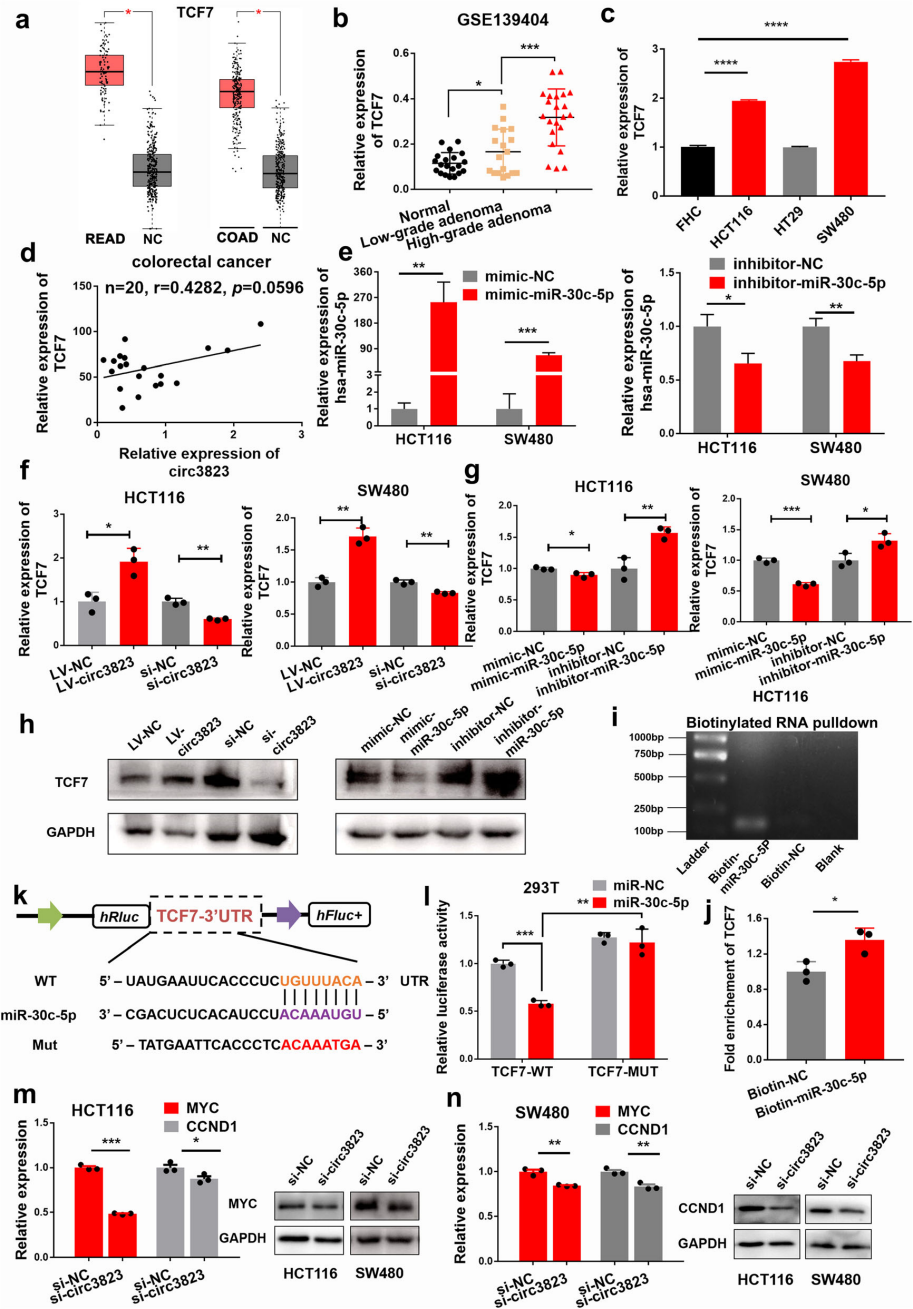
TCF7 is directly targeted by miR-30c-5p and indirectly regulated by circ3823. **a** TCF7 was significantly up-regulated in both rectal cancer and colon cancer according to GEPIA. **b** GEO data show that TCF7 is closely related to the histopathological grade of colon cancer. **c** The expression of TCF7 in CRC cells compared with FHC. **d** Pearson correlation analysis of circ3823 and TCF7 expression in 20 CRC tissues. **e** The mimic and inhibitor efficiency of hsa-miR-30c-5p in HCT116 and SW480. **f, g, h** Relative mRNA and protein levels of TCF7 were detected in cells after transfected with LV-NC, LV-circ3823, si-NC, si-circ3823, mimic-NC, mimic-miR-30c-5p, inhibitor-NC and inhibitor-miR-30c-5p using qRT-PCR and western blot, respectively. **i, j** RNA pull-down was executed in HCT116 cells, followed by qRT-PCR and nucleic acid electrophoresis to detect the enrichment of TCF7. **k** Schematic illustration of TCF7-WT and TCF7-Mut luciferase reporter vectors. **l** The relative luciferase activities were detected in 293 T cells after transfection with TCF7-WT or TCF7-Mut and miR-30c-5p mimics or mimic-NC, respectively. **m, n** Relative expression of TCF7 downstream molecules MYC and CCND at RNA and protein level in cells transfected with si-circ3823. Data were indicated as mean ± SD, ns *P* ≥ 0.05, *P < 0.05, **P < 0.01, ***P < 0.001, ****P < 0.0001

According to the TargetScan, TCF7 and circ3823 share the same miRNA response elements (MRE) of miR-30c-5p. Then, we inquired through the GEPIA website that TCF7 was significantly highly expressed in both rectal cancer and colon cancer (Fig. 6a). In addition, GEO data showed that TCF7 was closely related to the histopathological grade of colon cancer. In normal tissues, low-grade adenomas and high-grade adenomas, the expression level of TCF7 gradually increased (Fig. 6b). Next, we detected the expression of circ3823 in CRC cell lines (HCT116, HT29, SW480), normal colorectal mucosal cells (FHC) and 20 pairs of CRC tissues by qRT-PCR. The results showed that compared with FHC, TCF7 was significantly higher expressed in HCT116 and SW480 cell lines (Fig. 6c). In addition, in 20 pairs of tissue samples, TCF7 and circ3823 showed potential of correlation (Fig. 6d).

Next, we knocked down or overexpressed circ3823 in HCT116 and SW480 cell lines (Fig. 3a). TCF7 decreased with the knockdown of circ3823 and increased with the overexpression of circ3823 (Fig. 6f). Similarly, we transfected mimic or inhibitor of miR-30c-5p in HCT116 and SW480 cell lines (Fig. 6e). The mimic of miR-30c-5p significantly reduced the mRNA and protein levels of TCF7, and the inhibitor of miR-30c-5p increased the mRNA and protein levels of TCF7 in HCT116 and SW480 cells (Fig. 6g, h).

MicroRNA pull-down assay were performed to verify the binding of TCF7 and miR-30c-5p in HCT116. The specific enrichment of TCF7 was detected by qRT-PCR in the miR-30c-5p probe group compared with control probe (Fig. 6i, j). In addition, dual luciferase reporter assay showed that the activity of luciferase reporter vector carrying the TCF7 3’UTR-WT sequence could be significantly decreased by miR-30c-5p mimics (Fig. 6k, l). As we all know, TCF7 is a key transcription factor in Wnt pathway. The increase of TCF7 is beneficial to activate the downstream functional molecules MYC and CCND1. Knock down circ3823 significantly reduced the mRNA and protein levels of MYC and CCND1 (Fig. 6m, n). These data suggest that circ3823 regulates the expression of TCF7 and downstream molecules MYC and CCND1 through serving as a ceRNA for miR-30c-5p in CRC.

**Fig. 7:**
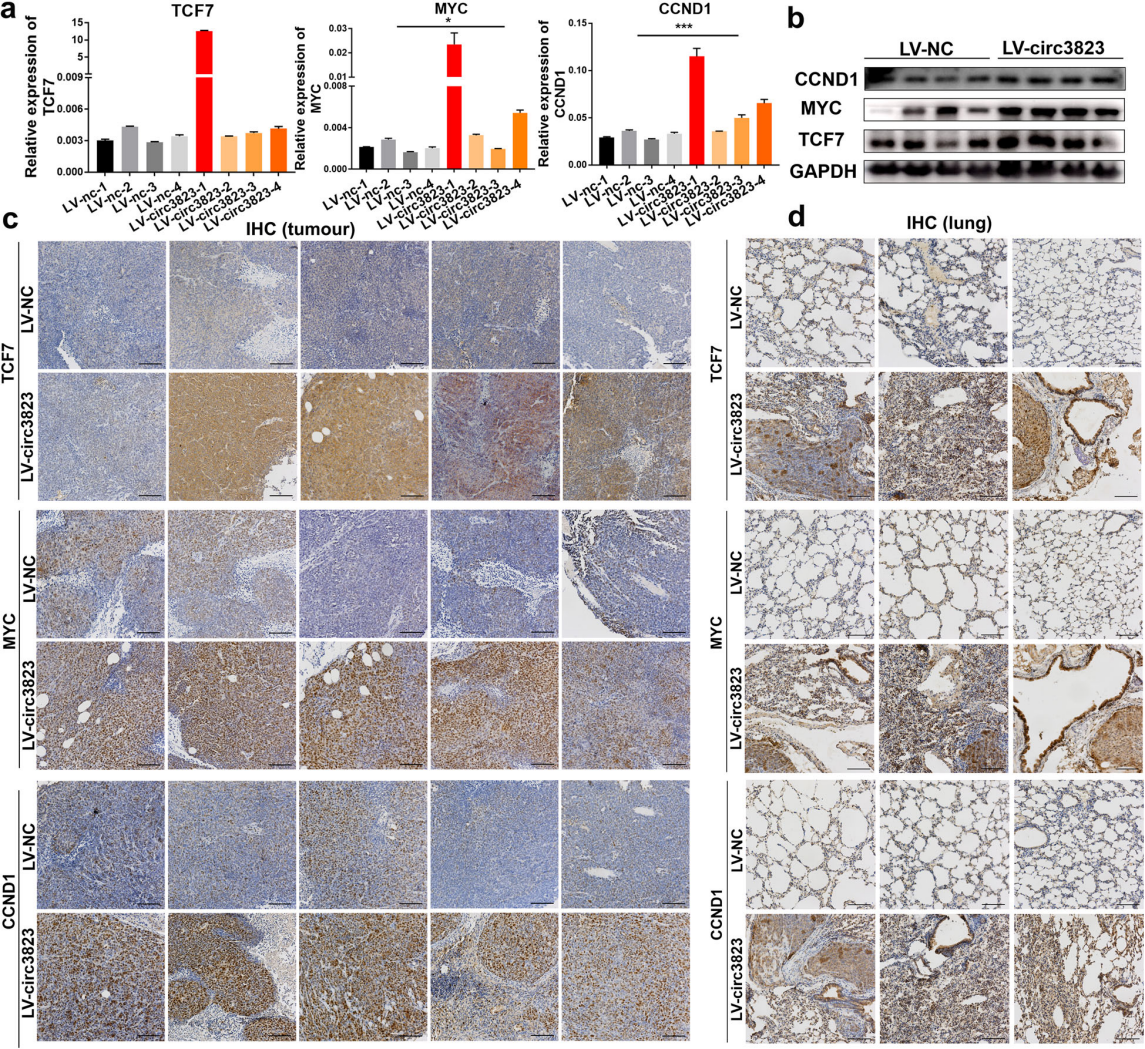
The RNA and protein levels of TCF7, MYC, CCND1 in subcutaneous xenograft tumours and lung metastatic tissues in nude mice. **a, b** Relative mRNA and protein levels of TCF7, MYC, CCND1 were detected in subcutaneous xenograft tumours by qRT-PCR and western blot. **c, d** Relative protein levels of TCF7, MYC, CCND1 were detected in subcutaneous xenograft tumours and lung metastatic tissues by ISH (magnification, × 100, scale bar, 100 μm). Data were indicated as mean ± SD, ns *P* ≥ 0.05, *P < 0.05, **P < 0.01, ***P < 0.001, ****P < 0.0001

The in vivo experimental results are consistent with the in vitro results. Compared with the LV-NC group, the mRNA and protein levels of MYC and CCND1 were significantly higher in the LV-circ3823 group (Fig. 7a, b). In addition, we used nude mouse subcutaneous tumours and lung metastases to make paraffin sections and perform IHC experiments. IHC results showed that the positive cell rates of TCF7, MYC, and CCND1 were significantly higher in the LV-circ3823 group compared with LV-NC group (Fig. 7c, d).

### Circ3823 promotes CRC cell proliferation and metastasis through circ3823/miR-30c-5p/TCF7 axis

**Fig. 8:**
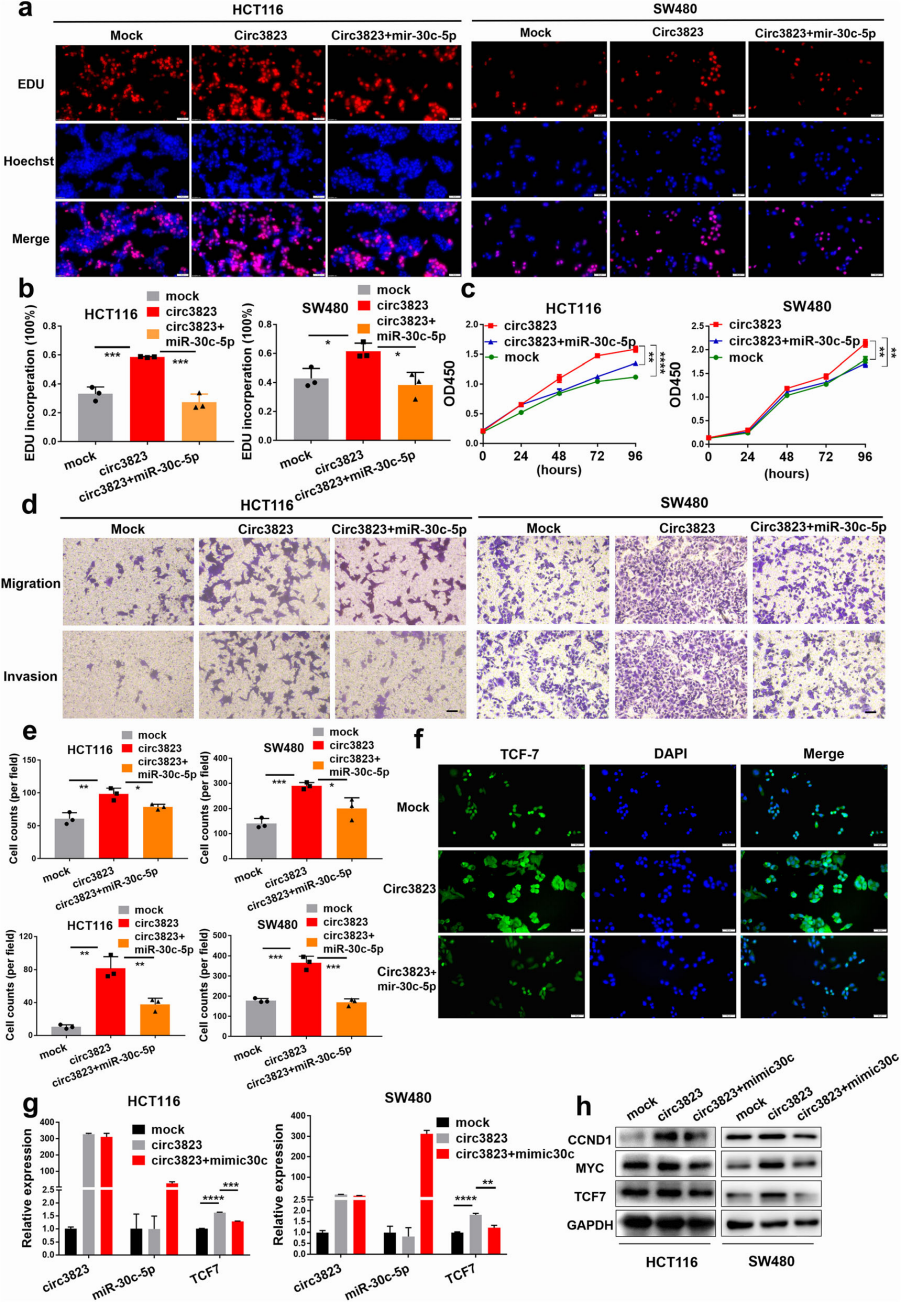
Circ3823 promotes cell proliferation and metastasis through circ3823/miR-30c-5p/TCF7 axis. **a, b, c** The cell proliferation were determined after transfection with overexpression circ3823 vectors and mimic-miR-30c-5p by EdU and CCK-8 assays (magnification, × 200, scale bar, 50 μm). **d, e** The cell migration and invasion were determined after transfection with overexpression circ3823 vectors, and mimic-miR-30c-5p by trans well assays (magnification, × 100, scale bar, 100 μm). **f** Relative protein levels of TCF7 in cells were assessed by IF after transfection (magnification, × 200, scale bar, 50 μm). **g, h** Relative expression of TCF7 mRNA and protein levels were detected by qRT-PCR and western blot in cells transfected with overexpression circ3823 vectors and mimic-miR-30c-5p. Data were indicated as mean ± SD, ns *P* ≥ 0.05, *P < 0.05, **P < 0.01, ***P < 0.001, ****P < 0.0001

**Fig. 9:**
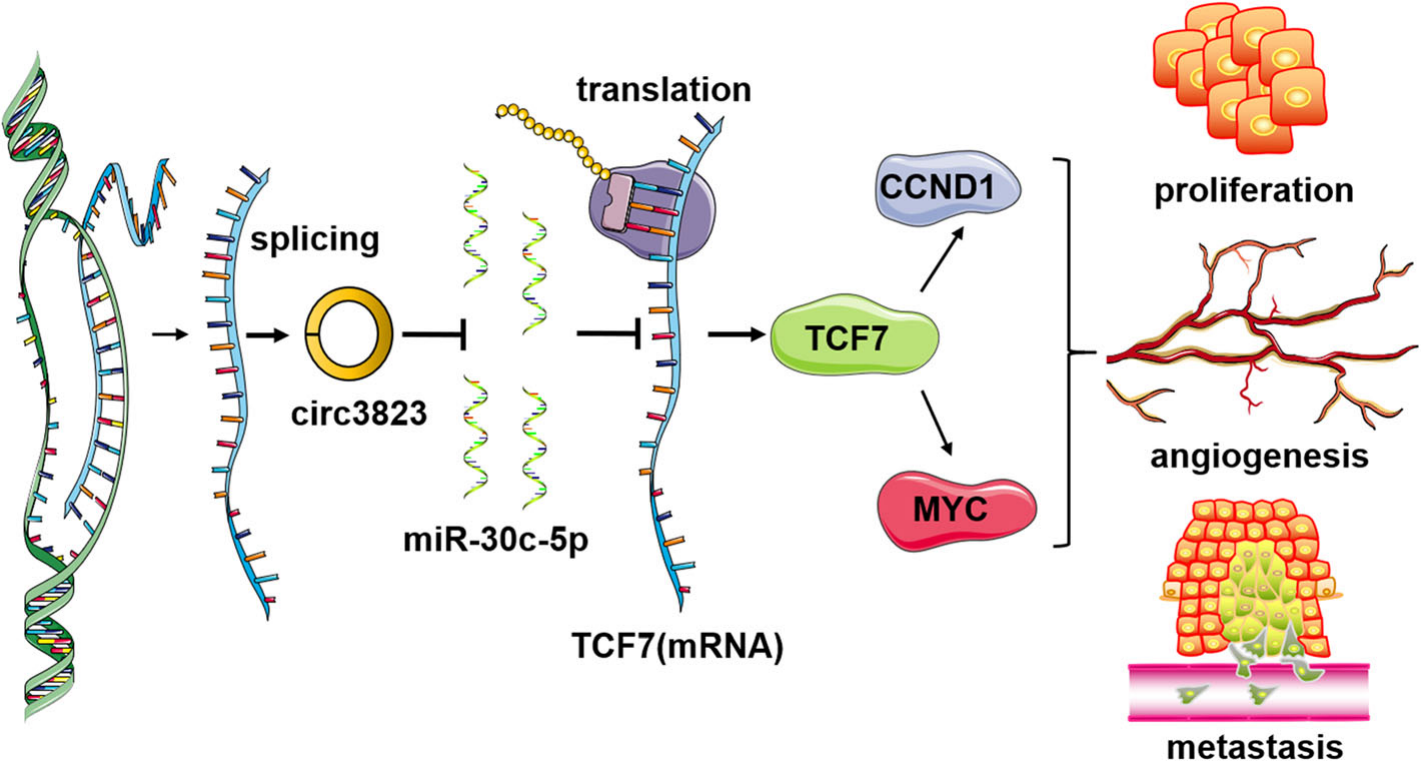
Schematic representation of a model for the major molecular mechanisms of “circ3823/miR-30c-5p/TCF7” axis in CRC

To explore whether circ3823 serves its biological function through circ3823/miR–30c-5p/TCF7 axis, rescue experiments were designed using miR-30c-5p mimics and inhibitors. EdU, CCK8 and transwell assays indicated that the miR-30c-5p mimics reversed the proliferation, migration and invasion-promoting effects induced by overexpression of circ3823 in HCT116 and SW480 cells (Fig. 8a, b, c, d, e). IF staining displayed that overexpression of circ3823 significantly increased the levels of TCF7 and the effect could be abolished by miR-30c-5p mimics (Fig. 8f). Furthermore, qRT-PCR and western blot demonstrated that upregulation of circ3823 enhanced the mRNA and protein levels of TCF7 and downstream targets MYC and CCND1 and the effects caused by overexpressing circ3823 could be reversed by miR-30c-5p mimics (Fig. 8g, h). In summary, these data demonstrated that circ3823 might serve as a ceRNA for miR-30c-5p to regulate TCF7 expression, which led to proliferation, migration and invasion and development of CRC (Fig. 9).

### N6-methyladenosine modification exists on circ3823 and affects its expression level

According to Methylated RNA immunoprecipitation (MeRIP), anti-m6A antibodies specifically enrich circ3823 (Fig. S2a). This indicates that the N6-methyladenosine methylation modification site exists on circ3823. Besides, circ3823 was significantly highly expressed in HCT116 cell after transfection with si-YTHDF3 and si-ALKBH5 compared with the control group (Fig. S2b, c). In addition, YTHDF3 and ALKBH5 were significantly reduced in CRC according to GEO and TCGA (Fig. S2d, e, f). Furthermore, YTHDF3 and ALKBH5 were positively correlated with YTHDF2 according to TCGA COAD data (Fig. S2g). These results indicate that the degradation of circ3823 may be regulated by m6A recognition protein YTHDF3 and demethylase ALKBH5.

## Discussion

CircRNA expression profiling is a prerequisite for the identification of novel oncogenic circRNAs and tumour suppressors, as well as in elucidating their mechanisms and functions [30]. Here, we applied RNA-seq to obtain the expression profiles of circRNA as well as mRNA in CRC tissues and normal tissues. Subsequently, we identified a novel circRNA termed circ3823 which was obviously highly expressed in CRC tissues. And particularly valuable discovery is the expression of circ3823 in serum has high sensitivity and specificity for detecting CRC which means circ3823 has the potential to be used as liquid diagnostic biomarker. Liquid biopsy refers to the analysis of tumours using biomarkers circulating in liquids such as blood, urine, ascites and cerebrospinal fluid [31]. Circulating tumour cells, exosomes, and nucleic acids (DNA and RNA) have become attractive candidates for liquid biopsy because they have many key characteristics of ideal biomarkers [32]. The ability to detect and characterize tumours in this minimally invasive and reproducible manner has considerable clinical significance [33]. Due to the non-invasive and low-cost advantages of liquid diagnostics, circ3823 have the potential to be used for large-scale population screening of CRC in the future. GO functional annotation and KEGG enrichment analysis showed that the enriched function and pathway were related to apparent characteristics of CRC. The apparent characteristics of CRC include: tumour cell proliferation, apoptosis, infiltration and migration, stromal cell adhesion, angiogenesis, and tumour immunity, etc. Among them, angiogenesis is an indispensable inducement for tumour cell growth and metastasis. Our experiments confirmed that circ3823 significantly increase tumour neovascularization both in vivo *and* in vitro.

The classic regulatory mechanisms of circRNA include ceRNA, RNA-binding proteins and functional proteins coding. Among these mechanisms, ceRNA hypothesis represents complex post-transcriptional regulatory network. Growing evidences indicated that circRNA regulates mRNA expression by competing for shared MRE [34]. For example, circCRIM1 upregulating FOXQ1 to promote metastasis and docetaxel chemoresistance of nasopharyngeal carcinoma [35]. Besides, circ-Erbin mediated HIF-1α activation by miR-125a-5p/4EBP-1 axis [13]. Moreover, circSOD2 inhibits miR-502-5p expression and rescues the target gene DNMT3a [36]. In this study, circ3823 exerted its function as a ceRNA that competitively bound to miR-30c-5p, then abolished the endogenous suppressive effect of miR-30c-5p on the target gene TCF7.

TCF7 is a key transcriptional effector of Wnt signaling pathway, which plays a very important regulatory role in tumorigenesis and development. TCF7 protein was identified to be highly expressed in various cancers, such as lung cancer, pancreas cancer, and breast carcinomas [37–40]. In this study, we verified that TCF7 was highly expressed in CRC tissues. Circ3823 significantly increases both the mRNA and protein levels of TCF7 in CRC. In addition, elevated TCF7 promote the expression of MYC and CCND1 in CRC, leading to activation of Wnt signaling pathway. Revealing that circ3823 drive CRC cell proliferation, metastasis and angiogenesis depends on the highly expressed TCF7 in CRC.

We noticed that although the overexpression of circ3823 did not effectively introduce to increase the RNA levels of TCF7, MYC, and CCND1, those proteins were detected clearly in IHC of the xenograft. This result prompted us to think about the reasons for the discrepancy between RNA and protein levels. By consulting literatures, we learned that gene expression is divided into two levels, transcription and translation, that is mRNA level and protein level. First of all, there is a spatial and temporal interval between transcription and translation of eukaryotic genes [41, 42]. It is possible that the express of protein is still increasing when the mRNA reaches its peak, or that the mRNA is already degraded when the protein level reaches its peak. Secondly, after transcription, there is post-transcriptional processing, such as degradation of transcription products and post-translational modifications. Hence, the transcription level and translation level are not exactly the same [43–45]. In addition, there are negative feedback regulatory mechanisms in the organism, and the cells of the organism need to maintain homeostasis [46–48]. When protein levels are elevated, it is a stress for the cell which inevitably decreases gene transcription in order to maintain homeostasis in the body. Conversely, when protein levels are low, the cell itself may promote transcription.

Currently, most explorations focus on the downstream mechanism of circRNA in tumour progression. However, the upstream mechanisms of circRNA generation, splicing, and degradation are rarely explored. Increasing evidence indicates that m6A modification was involved in regulating mRNA degradation [49–53]. Therefore, we guessed whether circRNA degradation is also regulated by m6A modification? Previous research revealed that YTHDF2 recruits the adaptor protein HRSP12 and RNase P/MRP complex to the cleavage circRNA by reading m6A site. Resulting in the closed loop structure of circRNA was destroyed and rapidly degraded [54]. The most recent article firstly proposed that N6-methyladenosine modification at specific sites promote the stability of circRNA, but the specific mechanism has not been further explored [55]. Based on our results of RIP experiments, we found that m6A antibodies specifically enrich circ3823. Moreover, after transfection of si-YTHDF3 and si-ALKBH5, the expression of circ3823 was significantly up-regulated compared with the control group. In addition, according to TCGA and GEO database, we found that YTHDF3 and ALKBH5 were significantly reduced in CRC and YTHDF3/ALKBH5 were positively correlated with YTHDF2. By consulting literatures, we learned that YTHDF3 binds m6A in cells and shares mRNA targets with YTHDF2, YTHDF3 cooperate with YTHDF2 to promote mRNA degradation [50]. Therefore, we speculate whether YTHDF3 and ALKBH5 cooperate with YTHDF2 to promote the degradation of circ3823. The above conjecture needs to be verified by rigorous experiments, which is the subject of our next exploration.

## Conclusions

In conclusion, our results suggest that circ3823 evidently highly-expressed in tissues and serums of CRC. The sensitivity and specificity for detecting CRC means that circ3823 have the potential to be used as diagnostic biomarkers. Due to the non-invasive and low-cost advantages of liquid diagnostics, circ3823 have the potential to be used for large-scale population screening of CRC in the future. We firstly demonstrated that circ3823 might sponge miR-30c-5p to upregulate TCF7 expression, leading to proliferation, metastasis and angiogenesis of CRC. The angiogenesis effect of circ3823 is particularly significant in nude mice xenograft tumours. In addition, we found that the N6-methyladenosine modification may regulate the degradation of circ3823, and first put forward the hypothesis that YTHDF3 and ALKBH5 cooperate with YTHDF2 to promote the degradation of circ3823. Our findings provide an insight into understanding the progression of CRC, and provide potential therapeutic targets for CRC.

## Supplementary Information

**Additional file 1.**

## Data Availability

All the data obtained and/or analyzed during the current study were available from the corresponding authors on reasonable request.

## Abbreviations

CRC: Colorectal cancer
qRT-PCR: Quantitative reverse transcription-polymerase chain reaction
ROCs: Receiver operating characteristic curves
CircRNA: Circular RNA
ceRNA: Competing endogenous RNA
GEO: Gene Expression Omnibus
TCGA: The Cancer Genome Atlas
ISH: In situ hybridization
FISH: Fluorescent in situ hybridization
RIP: RNA immunoprecipitation
IHC: Immunohistochemistry
IF: Immunofluorescence
MRE: miRNA response elements
HE: Hematoxylin and Eosin
RISC: RNA-induced silencing complex
