# Supplementary material for: Circ3823 contributes to growth, metastasis and angiogenesis of colorectal cancer: involvement of miR-30c-5p/TCF7 axis
Source: Mol Cancer. 2021 Jun 25;20:93. doi: 10.1186/s12943-021-01372-0 (PMC8229759; doi:10.1186/s12943-021-01372-0)
Supplement: Supplementary file 1 — Additional file 1. [file 12943_2021_1372_MOESM1_ESM.docx]

**Supplementary materials**


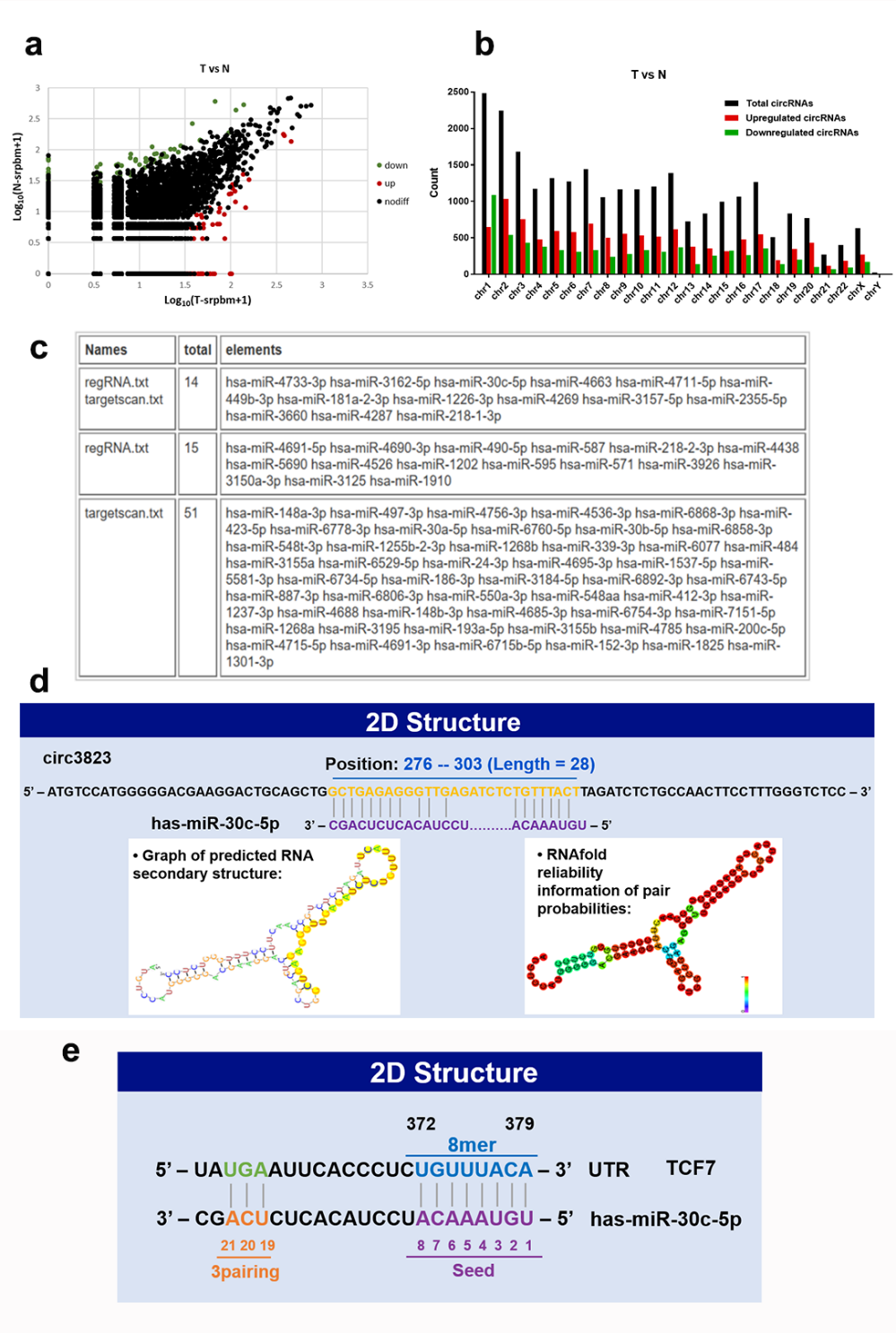


**Fig. S1** Statistical analysis of RNA-seq and 2D model diagram of circ3823/miR-30c-5P/TCF7 axis based on bioinformatics prediction. **a** Scatter plot showing the differential expression of circRNAs and sample correlation. **b** Distribution of circRNA on human chromosomes. **c** MicroRNAs screened based on the intersection of TargetScan and RegRNA 2.0. **d, e** 2D model diagram of circ3823/miR-30c-5P/TCF7 axis and predicted RNA secondary structure.


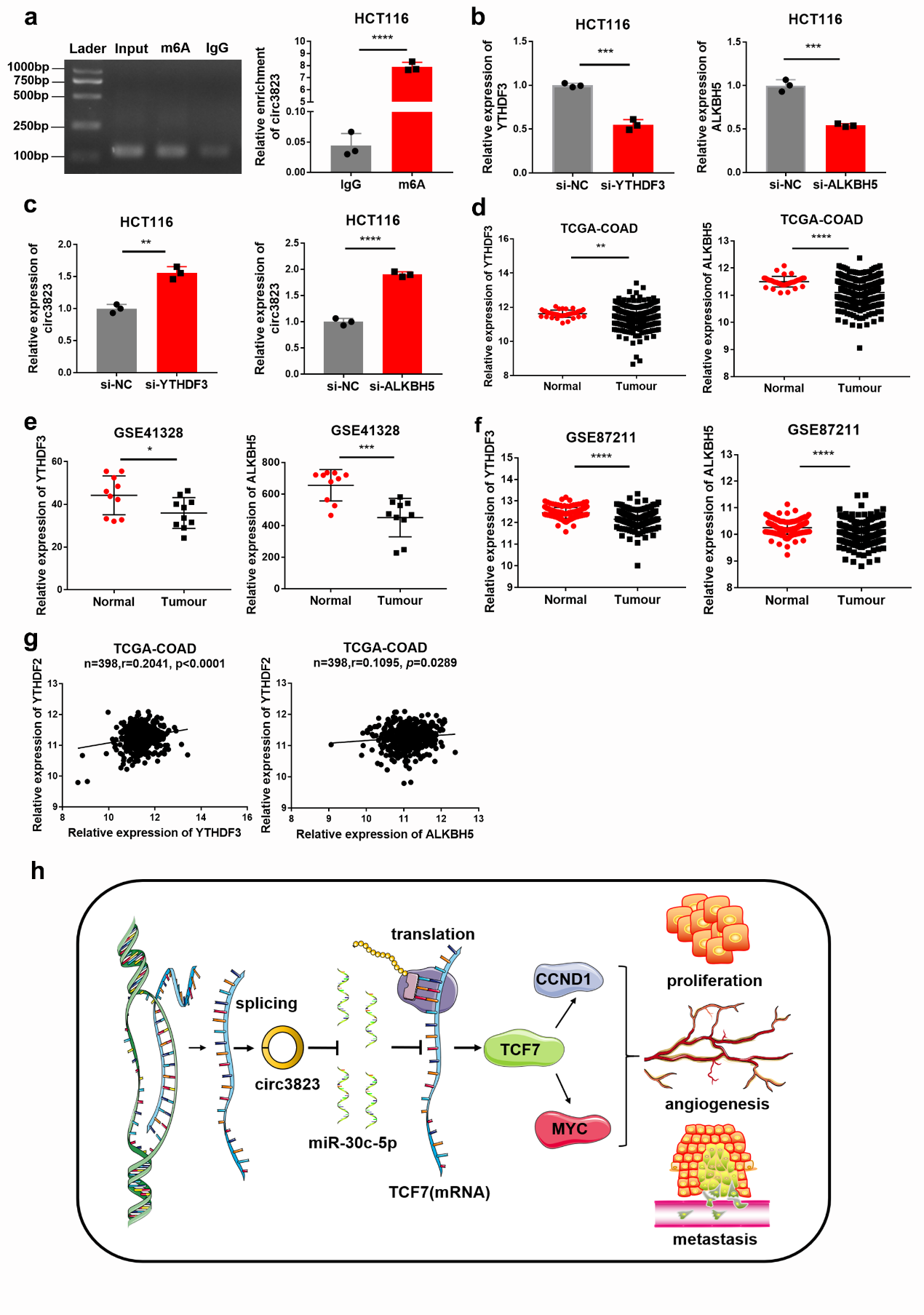


**Fig. S2:** N6-methyladenosine thrmethylation modification exists on circ3823 and may affects its expression. **a** m6A antibodies can specifically enrich circ3823. **b, c** The expression of circ3823 was significantly up-regulated in HCT116 cells after transfection with si-YTHDF3 and si-ALKBH5 compared with the control group. **d, e, f** YTHDF3 and ALKBH5 were significantly down-regulated in CRC according to GEO and TCGA. **g** YTHDF3 and ALKBH5 are positively correlated with YTHDF2 according to TCGA's COAD data. Data were indicated as mean ± SD, ns *P* ≥ 0.05, **P* < 0.05, ***P* < 0.01, ****P* < 0.001, *****P* < 0.0001.

Table S1： Personal characteristics of the patients who donated tissues for sequencing

| **Sex** | **Age** | **Tumour**  **type** | **Tumour differentiation** | **TNM**  **stage** | **Depth of tumour invasion** | **Metastasis** | **Previous history of CRC** |
| --- | --- | --- | --- | --- | --- | --- | --- |
| M | 49 | Adenocarcinoma | [Moderate differentiation](http://dict.cnki.net/dict_result.aspx?searchword=%e4%b8%ad%e4%bd%8e%e5%88%86%e5%8c%96&tjType=sentence&style=&t=moderate+or+poor+differentiation) | T1N0M0 | submucosa | No lymph node metastasis | None |
| F | 73 | Adenocarcinoma | [Moderate differentiation](http://dict.cnki.net/dict_result.aspx?searchword=%e4%b8%ad%e4%bd%8e%e5%88%86%e5%8c%96&tjType=sentence&style=&t=moderate+or+poor+differentiation) | T1N0M0 | submucosa | No lymph node metastasis | None |
| M | 50 | Adenocarcinoma | [Moderate](http://dict.cnki.net/dict_result.aspx?searchword=%e4%b8%ad%e4%bd%8e%e5%88%86%e5%8c%96&tjType=sentence&style=&t=moderate+or+poor+differentiation)  [differentiation](http://dict.cnki.net/dict_result.aspx?searchword=%e4%b8%ad%e4%bd%8e%e5%88%86%e5%8c%96&tjType=sentence&style=&t=moderate+or+poor+differentiation) | T1N0M0 | submucosa | No lymph node metastasis | None |
| F | 53 | Adenocarcinoma | [Moderate](http://dict.cnki.net/dict_result.aspx?searchword=%e4%b8%ad%e4%bd%8e%e5%88%86%e5%8c%96&tjType=sentence&style=&t=moderate+or+poor+differentiation)  [differentiation](http://dict.cnki.net/dict_result.aspx?searchword=%e4%b8%ad%e4%bd%8e%e5%88%86%e5%8c%96&tjType=sentence&style=&t=moderate+or+poor+differentiation) | T2N0M0 | shallow muscularis | No lymph node metastasis | None |

Table S2: Sequences of the qRT-PCR primers used in this study

| **Gene name** | **Forward primer (5’-3’)** | **Reverse primer (5’-3’)** |
| --- | --- | --- |
| circ3823 | CCCCGACTCTTCCTGGTGAA | CAGGCACAGCCATCTTGAGG |
| circ4953 | TGCACCACTTGGAACAGTTT | GCTGAGCCTGGCCACTATTT |
| circ2253 | TGGTGCACTTTCCTCCTTCT | AACATGGCAGTGACACCAAC |
| circ2749 | ATGCCTGTGAACCCATAGTGC | GAGCATCCCTATGGAGAGCAG |
| circ2990 | AAGGTACATCAAGGCACTGGC | CCCTGGATCACTGTTGGTCTG |
| circ3038 | TTTCATGCCGACTCTGACGA | GTCATCAGAGGATGACGAGGT |
| circ3651 | CAACGTTCAGTGCCTCGAAA | AACAGGCGGCTTAATGTGCT |
| circ8080 | AGCTTTCATGGCAGACCAGC | TACCGGAGCTCCTCAAAGGAA |
| TCF7 | CAAGCAGAGTCCAAGGCAGA | AGGATCTGGTTGATGGCAGC |
| MYC | TTCGGGTAGTGGAAAACCAG | CAGCAGCTCGAATTTCTTCC |
| CCND1 | GTCGCTGGAGCCCGTGAA | GGCCGGATGGAGTTGTCG |
| BCL2 | TGGGAGGAAAAGAGTTGGGA | AGTATTGGGAGTTGGGGGGT |
| BAX | TCTGACGGCAACTTCAACTGGG | CGCCACAAAGATGGTCACGG |
| Caspase3 | AGGAGCAGTTTTGTTTGTGT | AGTTTGGGTTTTCCAGTTAG |
| Caspase8 | CTACCAACTCATGGACCACA | CTGACAAGCCTGAATAAAAA |
| Caspase9 | AACCCTAGAAAACCTTACCC | ATCACCAAATCCTCCAGAAC |
| YTHDF3 | ATGGTAATAAGCGTTTGGATG | TTCTAAGCGAATATGCCGTAA |
| ALKBH5 | TGGAGATGGACAAGGAAGAGAA | AAAACCCCCCAATGAACAAA |
| GAPDH | AACGGATTTGGTCGTATTGG | TTGATTTTGGAGGGATCTCG |

Table S3: Sequences of the divergent and convergent primers used in this study

|  | **Gene name** | **Forward primer (5’-3’)** | **Reverse primer (5’-3’)** |
| --- | --- | --- | --- |
| **Convergent** | circ3823 | AGGGAGGCTGTGGCTGAA | GGGAGACCCAAAGGAAGTT |
| **Divergent** | circ3823 | CCCCGACTCTTCCTGGTGAA | CAGGCACAGCCATCTTGAGG |

Table S4: Sequences of the ISH, FISH and pulldown probes used in this study

| **Gene name** | **Probe (5’-3’)** |
| --- | --- |
| circ3823 (ISH) | DIG-AAAGATCAGGCCTCAAGCCCAGCT-DIG |
| circ3823 (FISH) | Cy3-GAAAAGATCAGGCCTCAAGCCCAGCTG |
| miR-30c-5p (FISH) | FAM-GCTGAGAGTGTAGGATGTTTACA |
| miR-30c-5p pulldown probe | UGUAAACAUCCUACACUCUCAGC-Biotin |
| NC pulldown probe | UUGUACUACACAAAAGUACUG-Biotin |

Table S5: Sequences of the siRNA used in this study

| **Gene name** | **Probe (5’-3’)** |
| --- | --- |
| Si-circ3823-001 | GCTTGAGGCCTGATCTTTT |
| Si-circ3823-002 | TTGAGGCCTGATCTTTTGG |
| Si-circ3823-003 | CTTGAGGCCTGATCTTTTG |
| Si-YTHDF3 | CATACATCGTTCCATTAAA |
| Si-ALKBH5 | GATCGCCTGTCAGGAAACA |
